# Thermoneutrality decreases thermogenic program and promotes adiposity in high‐fat diet‐fed mice

**DOI:** 10.14814/phy2.12799

**Published:** 2016-05-26

**Authors:** Xin Cui, Ngoc Ly T. Nguyen, Eleen Zarebidaki, Qiang Cao, Fenfen Li, Lin Zha, Timothy Bartness, Hang Shi, Bingzhong Xue

**Affiliations:** ^1^ Department of Biology and Center for Obesity ReversalGeorgia State UniversityAtlantaGeorgia

**Keywords:** Beige adipocytes, brown adipocytes, obesity, thermoneutrality, UCP1

## Abstract

Brown/beige adipocytes are therapeutic targets to combat obesity due to their abilities to dissipate energy through adaptive thermogenesis. Most studies investigating induction of brown/beige adipocytes were conducted in cold condition (e.g., 4°C); much is unknown about how the thermogenic program of brown/beige adipocytes is regulated in thermoneutral condition (e.g., 30°C), which is within the thermal comfort zone of human dwellings in daily life. Therefore, this study aims to characterize the thermogenic program of brown/beige adipocytes in mice housed under ambient (22°C) versus thermoneutral condition (30°C). Male mice raised at 22°C or 30°C were fed either chow diet or high‐fat (HF) diet for 20 weeks. Despite less food intake, chow‐fed mice housed at 30°C remained the same body weight compared to mice at 22°C. However, these thermoneutrally housed mice displayed a decrease in the expression of thermogenic program in both brown and white fat depots with larger adipocytes. When pair‐fed with chow diet, thermoneutrally housed mice showed an increase in body weight. Moreover, thermoneutrality increased body weight of mice fed with HF diet. This was associated with decreased expression of the thermogenic program in both brown and white fat depots of the thermoneutrally housed mice. The downregulation of the thermogenic program might have resulted from decreased sympathetic drive in the thermoneutrally housed mice evident by decreased expression of tyrosine hydroxylase expression and norepinephrine turnover in both brown and white fat depots. Our data demonstrate that thermoneutrality may negatively regulate the thermogenic program and sympathetic drive, leading to increased adiposity in mice.

## Introduction

Obesity has become a world‐wide epidemic problem, and is an independent risk factor for a panel of metabolic diseases, including insulin resistance/type 2 diabetes, dyslipidemia, cardiovascular diseases, and certain types of cancer. Therefore, it is important to better understand the mechanisms regulating energy homeostasis and to identify pathways that can intervene the etiology of obesity. Obesity develops when a persistent imbalance between energy intake and energy expenditure occurs (Hill et al. 2012). Energy expenditure is the sum of energy expended to maintain physiological and metabolic processes, to perform work and to generate heat. The last is collectively called adaptive thermogenesis, which includes shivering and nonshivering thermogenesis. While shivering usually occurs in the muscle, nonshivering thermogenesis primarily occurs in brown adipose tissue (BAT) (Donahoo et al. 2004; Hill et al. 2012). This is due to the unique expression of uncoupling protein 1 (UCP1) in the inner mitochondrial membrane, which serves to uncouple oxidative phosphorylation from ATP synthesis, thereby profoundly increase energy expenditure (Nicholls and Locke 1984; Cannon and Nedergaard 1985).

In rodents, there exist two types of UCP1‐expressing adipocytes (Seale et al. 2008; Ishibashi and Seale 2010; Petrovic et al. 2010; Wu et al. 2012). Traditional brown adipocytes are located in discrete areas, such as the interscapular BAT; whereas “inducible” beige adipocytes are dispersed in white adipose tissue (WAT) (Seale et al. 2008; Ishibashi and Seale 2010; Petrovic et al. 2010; Wu et al. 2012), and can be induced by *β*3‐adrenergic receptor activation or cold exposure (Himms‐Hagen 1989; Guerra et al. 1998; Xue et al. 2005) through activation of the sympathetic nervous system (SNS) as measured neurochemically by norepinephrine turnover (NETO) (Bowers et al. 2004; Brito et al. 2007, 2008; Vaughan et al. 2014). It has long been reported that activation of brown/beige adipocyte thermogenesis alleviates obesity and its associated metabolic diseases (Himms‐Hagen et al. 1994; Guerra et al. 1998; Cederberg et al. 2001; Feldmann et al. 2009; Seale et al. 2011; Cohen et al. 2014). For example, activation of brown/beige adipocyte thermogenesis pharmacologically by administration of *β*3 adrenergic receptor agonist or genetically by overexpressing UCP1 in WAT relieves obesity and metabolic disorders in obese animal models (Himms‐Hagen et al. 1994; Kopecky et al. 1995, 1996); whereas ablation of UCP1 in mice housed under thermoneutral temperature results in obesity (Feldmann et al. 2009). Most importantly, recent discovery of functional brown and beige adipocytes in humans suggest that increasing brown/beige adipocyte thermogenic function may be a novel and promising approach in treating obesity (Cypess et al. 2009; Marken Lichtenbelt et al. 2009; Virtanen et al. 2009).

The amount of energy that is used for thermogenesis is strongly dependent on the environmental temperature and decreases with increasing temperature as the heat generation demand for maintaining body temperature decreases, until a critical temperature is reached above which no extra heat need to be generated to maintain body temperature. That critical temperature is where the thermoneutral window starts (Speakman and Keijer 2012; Lichtenbelt et al. 2014). Thermoneutrality is the temperature where energy expenditure to maintain body temperature is minimal (Gordon 1990; Lichtenbelt et al. 2014). Instead, normal ambient temperature (22°C) where most mice are housed in current studies causes a chronic thermal stress to mice. To defend their body temperatures, the mice must increase their energy expenditure to generate heat via adaptive thermogenesis (Chaffee and Roberts 1971; Cannon and Nedergaard 2004), which significantly contributes to the total energy expenditure of the mice (Golozoubova et al. 2004; Ravussin et al. 2012). Thus, the constant thermal stress of the animal‐housing conditions may have a significant impact on the outcomes of earlier metabolic studies (Kozak and Anunciado‐Koza 2008; Feldmann et al. 2009; Overton 2010; Cannon and Nedergaard 2011). Recently, more studies on energy metabolism have been conducted under thermoneutrality, where thermal stress was minimal (Keith et al. 2006; Feldmann et al. 2009; McAllister et al. 2009; Hansen et al. 2010; Johnson et al. 2011). Moreover, most studies investigating induction of brown/beige adipocyte in rodents were conducted in the condition of cold (e.g., 4°C). However, the metabolic consequences of thermoneutrality on the thermogenic program of brown/beige adipocytes and obesity are less clear.

In developed countries, the ambient temperatures inside most dwellings and buildings are adjustable for people to get thermocomforts as they desire (Lichtenbelt et al. 2014). van Marken Linchtenbelt recently indicated that a slighter lower ambient temperature (e.g., 15–16°C) through cold acclimation can induce beige adipocyte appearance and enhance nonshivering thermogenesis in humans, which may play an important role in prevention of obesity (van der Lans et al. 2013; Lichtenbelt et al. 2014). However, much is unknown about how the thermogenic program of brown/beige adipocytes is regulated in the thermoneutral condition (e.g., 30°C), where loss of heat to the environment is marginal and energy demanded for regulatory thermogenesis against cold is supposedly suppressed (Lichtenbelt et al. 2014). Since the thermoneutral temperature is within the thermal comfort zone of human dwellings in daily life (Lichtenbelt et al. 2014), this study aims to determine the impact of thermoneutrality (30°C) on brown/beige adipocyte thermogenic program and obesity in mice. We housed the mice at either an ambient temperature (22°C) or thermoneutral temperature (30°C) and characterized the thermogenic program of brown/beige adipocytes and metabolic phenotypes under these thermal conditions.

## Materials and Methods

### Animals

All animal studies were approved by the Institutional Animal Care and Use Committee of the Georgia State University. Eight‐week‐old male and female A/J mice were purchased from Jackson Laboratories (Bar Harbor, ME), and housed in rooms with the ambient temperature set at either 22°C or 30°C. Breeding pairs were set at 22°C or 30°C to get pups, which were raised at the same temperature. Male mice housed at 22°C or 30°C were fed either chow diet or high‐fat diet (HFD, 60% calories from fat; Research Diets, Inc., New Brunswick, NJ) for 20 weeks. Animals were housed in polypropylene cages with a 12‐h light/dark cycle. The animals had free access to water and food. Body weight was measured every week. At the end of the studies, all animals were killed. Tissues were collected, snap‐frozen in liquid nitrogen, and stored at −80°C or fixed in 10% neutral formalin for further experiments as described below.

### RNA isolation and quantitative real‐time PCR

Total RNA from adipose tissue was extracted using the Tri‐Reagent kit (Molecular Research Center, Cincinnati, OH), and gene expression was assessed by quantitative reverse transcription‐polymerase chain reaction (PCR) (ABI Universal PCR Master Mix; Thermo Fisher Scientific, Waltham, MA) using a Stratagene Mx3000P thermocycler (Stratagene, La Jolla, CA). The gene expression data were normalized to the housekeeping gene cyclophilin. The primer and probe sets used in the assays were purchased from Thermo Fisher Scientific (Waltham, MA).

### Immunoblotting

Mouse adipose tissue homogenate (50 *μ*g protein extracted by RIPA solution) was subjected to electrophoresis on 4–16% gradient polyacrylamide gels (Criterion precast gel; Bio‐Rad Laboratories Inc., Hercules, CA), transferred to polyvinylidene fluoride membranes, incubated in blocking buffer (5% non‐fat dry milk in TBS) and then incubated with primary antibody UCP1 (Abcam, Cambridge, MA) (1:500 dilution) or Tyrosine Hydroxylase (Cell Signaling, Beverly, MA) (1:1000 dilution), washed with TBST, and incubated with fluorescence‐conjugated secondary antibody (1:5000 dilution). The blots were developed with a Li‐COR Imager System (Li‐COR Biosciences, Lincoln, NE) and normalized with the control *α*‐Tubulin (Cell Signaling, Beverly, MA) (1:500 dilution).

### Immunohistochemistry

Adipose tissues were fixed in 10% neutral formalin, embedded in paraffin, and cut into 5 *μ*m sections. Sections were processed for hematoxylin and eosin staining, and histological images were recorded using Nikon Eclipse E800 Microscopy with Zeiss AxioCam camera. Immunodetections were performed with UCP1 antibody (Santa Cruz Biotechnology, Dallas, TX) to examine UCP1‐positive cells in both brown and white adipose tissues.

### Food consumption and pair‐feeding experiment

For food consumption measurement, mice were caged individually. The amount of normal diet consumed was carefully monitored at 10:00 am local time every morning for a period of 1–2 weeks.

For pair‐feeding experiment, 8‐week‐old male A/J mice were purchased from Jackson Laboratories (Bar Harbor, ME), and housed at 30°C to acclimate for 3 weeks, all mice were single‐housed in regular cages, and had access to water ad libitum. Mice housed under the thermoneutral condition (30°C) were set as the control. During the pair‐feeding experiment, the control mice in the thermoneutral room were provided with sufficient food and food weight was measured in the morning at a fixed time to determine daily food intake within the last 24 h. The pair‐fed mice housed in the ambient room temperature (22°C) were fed at the onset of dark cycle with the same amount of food that the control mice ate on the previous day.

### Glucose and insulin tolerance tests

For glucose tolerance tests (GTTs), mice were fasted overnight (∼16 h) and the blood glucose levels were measured before and after 15, 30, 60, 90, and 120 min intraperitoneal (i.p.) injection of glucose (1 g/kg body weight) using OneTouch Ultra Glucose Meter (LifeScan, Milpitas, CA). For insulin tolerance tests (ITTs), food was removed for 4–6 h and mice were injected intraperitoneally with 1 U/kg body weight of insulin (Humulin R, Eli Lilly, Indiana, IN). Blood glucose levels were then measured at 0, 15, 30, 60, 90, and 120 min.

### Norepinephrine turnover

Norepinephrine turnover (NETO) was measured using the *α*‐methyl‐p‐tyrosine (AMPT) method as previously described (Nguyen et al. 2014). AMPT is a competitive inhibitor of tyrosine hydroxylase, the rate‐limiting enzyme in catecholamine biosynthesis. After AMPT administration, the endogenous levels of NE in the tissues decline at a rate proportional to the initial NE concentrations. Half of the animals were killed to obtain basal NE content for subsequent calculation of NETO. The other half of the mice were injected intraperitoneally with AMPT (300 mg AMPT/kg body weight; 25 mg/mL) initially and 2 h later were subsequently injected with a supplemental dose of AMPT (150 mg/kg body weight) to ensure the maintenance of tyrosine hydroxylase inhibition. These animals were then killed 4 h later after the first injection by rapid decapitation. Both brown and white adipose tissues were rapidly harvested, weighed, frozen in liquid nitrogen, and then stored at −80°C until assayed for the catecholamine contents.

The NE contents in tissues were measured using reverse‐phase HPLC with electrochemical detection. Briefly, tissue was thawed and homogenized in a solution containing dihydroxybenzylamine (DHBA, internal standard) in 0.2 mol/L perchloric acid (PCA) with 1 mg/ml ascorbic acid (AA). Following centrifugation for 15 min at 7500 × *g* at 4°C, catecholamines were extracted from the homogenate with alumina and were then eluted into PCA/AA. The catecholamines were measured using an ESA (Bedford, MA) HPLC system with electrochemical detection (Coulochem II). The mobile phase was Cat‐A‐Phase II, and the column was a HR‐80 reverse‐phase column. NETO was calculated in brown fat, subcutaneous fat, and epididymal fat depots as we previously described (Nguyen et al. 2014). Briefly, calculations were made according to the following formula: *k* = (lg[NE]_0_ − lg[NE]_4_)/(0.434 × 4) and *K* = *k*[NE]_0_, where k is the constant rate of NE efflux, [NE]_0_ is the initial NE concentration, [NE]_4_ is the final NE concentration, and *K* = NETO.

### Statistical analysis

All data are presented as means ± SE. Statistical comparison between groups was performed using Student's *t*‐test. A value of *P* < 0.05 was considered statistically significant.

## Results

### Thermoneutrality decreases the expression of the thermogenic program in brown and white fat

**Figure 1:**
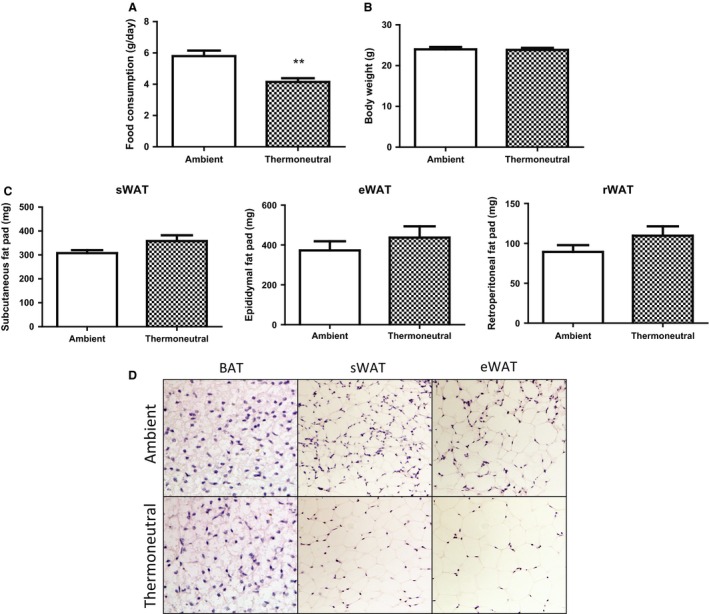
Thermoneutrality inhibits food intake but does not change body weight in chow‐fed mice. Mice under thermoneutrality had less food intake (A), while remained the same body weight (B) as under the ambient condition. (C–D) Thermoneutrality has a tendency to increase fat mass (C) and adipocyte size (D) in both BAT and WAT depots in chow‐fed mice. Mice were raised under either ambient (22°C) or thermoneutral (30°C) conditions. All data are expressed as mean ± SEM,* n* = 5; *P* < 0.05 versus Ambient control. BAT, brown adipose tissue; sWAT, subcutaneous white adipose tissue; eWAT, epididymal white adipose tissue; rWAT, retroperitoneal white adipose tissue.

**Figure 2:**
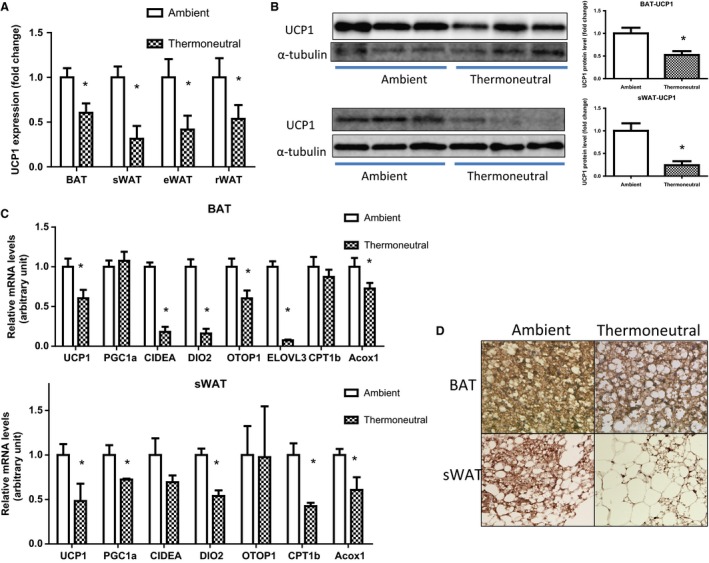
Thermoneutrality decreases the expression of the thermogenic program in both BAT and WAT of mice on chow diet. (A) Thermoneutrality decreases UCP1 mRNA in BAT and WAT depots. (B) Thermoneutrality decreases UCP1 protein in BAT and WAT depots. (C) Thermoneutrality decreases the mRNA expression of the thermogenic program in BAT and WAT depots. (D) Thermoneutrality decreases UCP1‐positive multiloccular cells in BAT and WAT depots. Mice were raised under either ambient (22°C) or thermoneutral (30°C) conditions. UCP1 mRNA and protein levels were measured by quantitative RT‐PCR and immunoblotting, respectively, and immunohistochemistry was used to examine UCP1‐positive cells, as described in the Materials and Methods. All data are expressed as mean ± SEM,* n* = 5; *P* < 0.05 versus Ambient control. BAT, brown adipose tissue; sWAT, subcutaneous white adipose tissue; eWAT, epididymal white adipose tissue; rWAT, retroperitoneal white adipose tissue.

Since the obesity‐resistant strain A/J mice have a higher expression of UCP1 and other thermogenic genes in white adipose tissue under normal ambient temperature (22°C) (Xue et al. 2005, 2007), we used this strain to study the impact of thermoneutrality (30°C) on the thermogenic program of brown/white fat, whose expression is expected to be further reduced. A/J breeders housed at 22°C or 30°C were set to breed pups, which were maintained under the same thermal condition after birth. Despite less food intake (Fig. 1A), the mice under the thermoneutral condition (30°C) remained the same body weight as the mice under the ambient condition (22°C) (Fig. 1B). However, we observed a tendency of increased fat mass in white fat depots such as subcutaneous fat (307.6 ± 12.6 mg vs. 358.0 ± 24.5 mg), epididymal fat (372.8 ± 45.9 mg vs. 437.0 ± 57.0 mg), and retroperitoneal fat (89.3 ± 8.6 mg vs. 109.7 ± 11.7 mg), with larger adipocytes in both brown (BAT) and white adipose tissue (WAT) of the thermoneutral group (Fig. 1C and D). Moreover, thermoneutrality significantly decreased UCP1 expression at both mRNA and protein levels in both brown fat and white fat depots such as subcutaneous fat, epididymal fat, and retroperitoneal fat (Fig. 2A and B). This was associated with downregulation of thermogenic gene expression in both BAT and WAT of the thermoneutral mice (Fig. 2C). The decreased expression of the thermogenic program was consistent with diminished UCP1‐positive multiloccular cells in both BAT and WAT of the thermoneutrally housed mice (Fig. 2D).

### Thermoneutrality promotes adiposity in mice given equal energy intake

**Figure 3:**
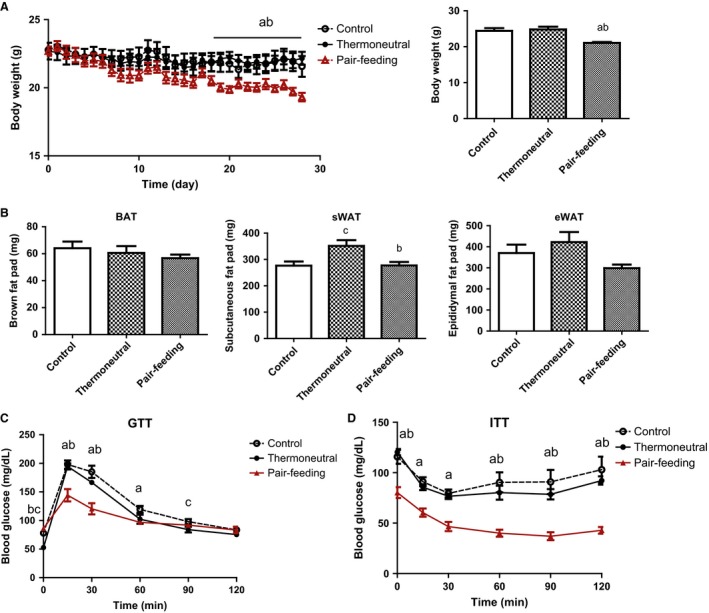
Thermoneutrality promotes adiposity and insulin resistance in a pair‐feeding experiment. Thermoneutrality increases body weight (A), fat mass (B), and causes insulin resistance in a pair‐feeding study. Mice were housed under either ambient (22°C) or thermoneutral (30°C) conditions for 3 weeks to acclimate. The pair‐feeding experiment, GTTs and ITTs were conducted as described in the Materials and Methods. All data are expressed as mean ± SEM,* n* = 6; a: pair‐feeding versus ambient control *P* < 0.05; b: thermoneutral versus pair‐feeding *P* < 0.05; c: thermoneutral versus the ambient control *P* < 0.05. sWAT, subcutaneous white adipose tissue; eWAT, epididymal white adipose tissue.

Since thermoneutrality decreased food intake by 30% (Fig. 1A), we therefore determined the impact of thermoneutrality on body weight and fat mass in a pair‐feeding study. Two‐month‐old male mice housed under either ambient (22°C) or thermoneutral (30°C) condition were used for pair‐feeding study, After pair‐fed with the same amount of food for 2 weeks, the mice housed at the ambient temperature (22°C) started showing a significant reduction of body weight, the difference of which was further diverged after 4 weeks, compared to the thermoneutrally housed mice (Fig. 3A). This was consistent with reduction of fat mass in the ambient mice pair‐fed with the same food intake (Fig. 3B). The reduction of adiposity was accompanied by improved glucose tolerance and insulin sensitivity assessed by GTTs and ITTs, respectively, in the mice housed at the ambient temperature (Fig. 3C and D).

### Thermoneutrality promotes adiposity in HF‐fed mice

**Figure 4:**
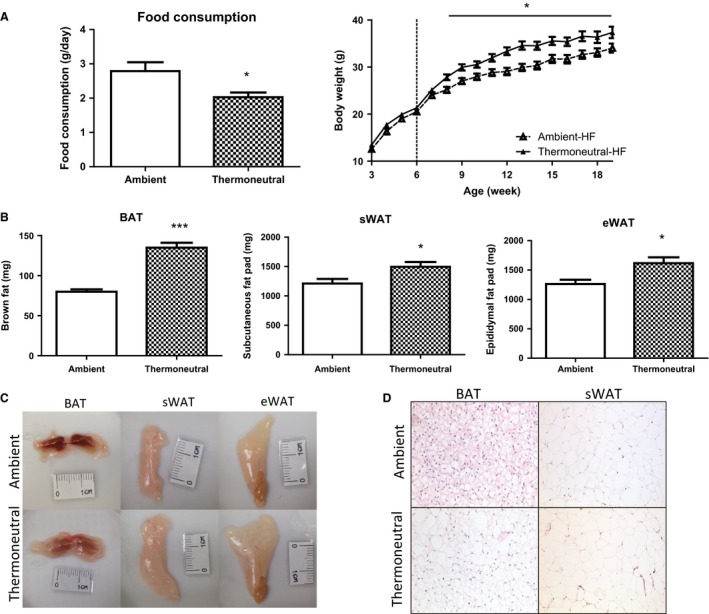
Thermoneutrality promotes adiposity in HF‐fed mice. Thermoneutrality, despite inhibiting food intake (A, left panel) promotes body weight (A, right panel), fat mass (B and C), and adipocyte size (D). Male mice raised at either 22°C or 30°C were fed high‐fat diet for 20 weeks. All data are expressed as mean ± SEM,* n* = 7; *P* < 0.05 versus Ambient control. BAT, brown adipose tissue; sWAT, subcutaneous white adipose tissue; eWAT, epididymal white adipose tissue.

**Figure 5:**
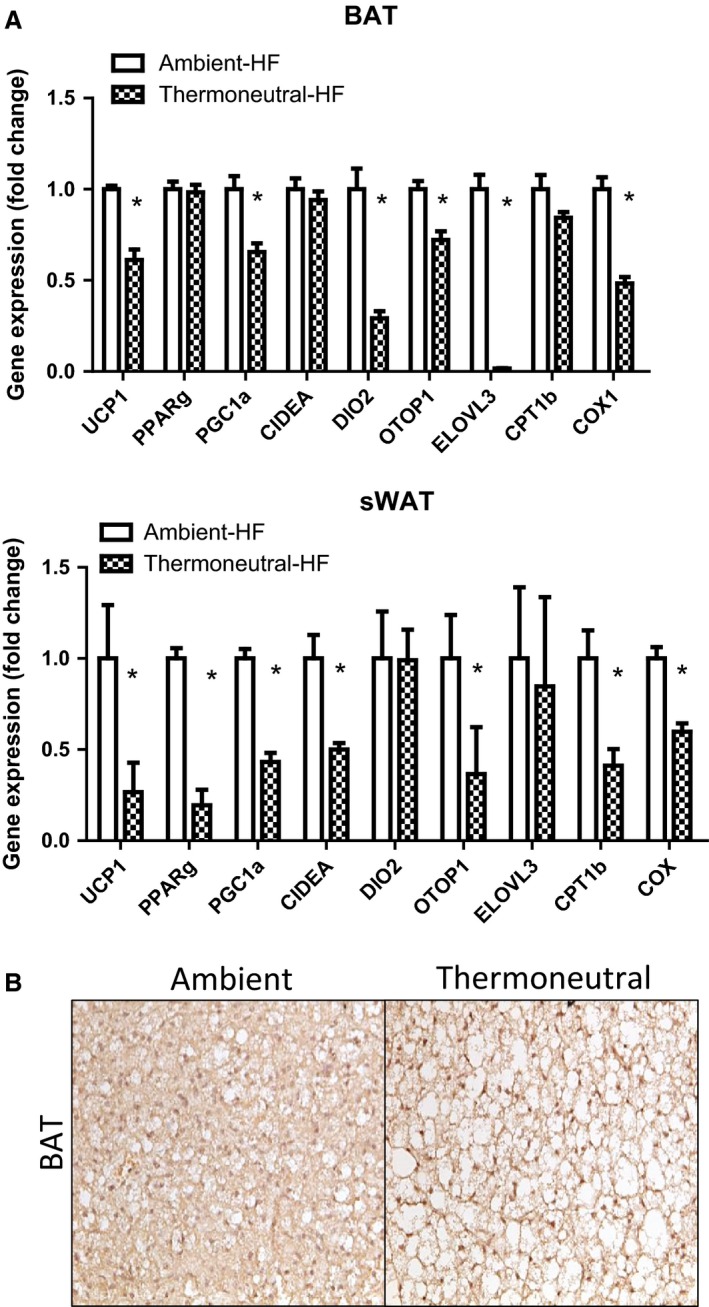
Thermoneutrality decreases the expression of the thermogenic program in both BAT and WAT of mice on HF diet. (A) Thermoneutrality decreases the mRNA expression of the thermogenic program in BAT and WAT depots. (B) Thermoneutrality decreases UCP1 protein staining in BAT depots. Male mice raised at either 22°C or 30°C were fed high‐fat diet for 20 weeks. mRNA levels of the thermogenic genes were measured by quantitative RT‐PCR and immunohistochemistry was used to examine UCP1 protein in adipocytes, as described in the Materials and Methods. All data are expressed as mean ± SEM,* n* = 7; *P* < 0.05 versus Ambient control. BAT, brown adipose tissue; sWAT, subcutaneous white adipose tissue.

We further determined the impact of thermoneutrality on the thermogenic program and adiposity in mice fed with HF diet. Male mice raised at either 22°C or 30°C were put on high‐fat diet at the age of 6 weeks. Despite eating less, mice housed under the thermoneutral condition (30°C) displayed increased body weight (Fig. 4A) as well as brown and subcutaneous white fat mass (Fig. 4B). In consistence, larger white fat and brown fat with paler color were observed in the mice housed at 30°C (Fig. 4C), and both BAT and WAT exhibited larger adipocytes (Fig. 4D). Moreover, thermoneutrality markedly suppressed the expression of the thermogenic program in both BAT and WAT of mice fed with HF diet (Fig. 5A). In consistence, our immunohistochemical analysis revealed a decrease in UCP1 protein staining in BAT (Fig. 5B). However, there was no change in glucose tolerance and insulin sensitivity between the mice housed under the ambient and the thermoneutral condition (data not shown).

### Thermoneutrality suppresses sympathetic nerve activity

**Figure 6:**
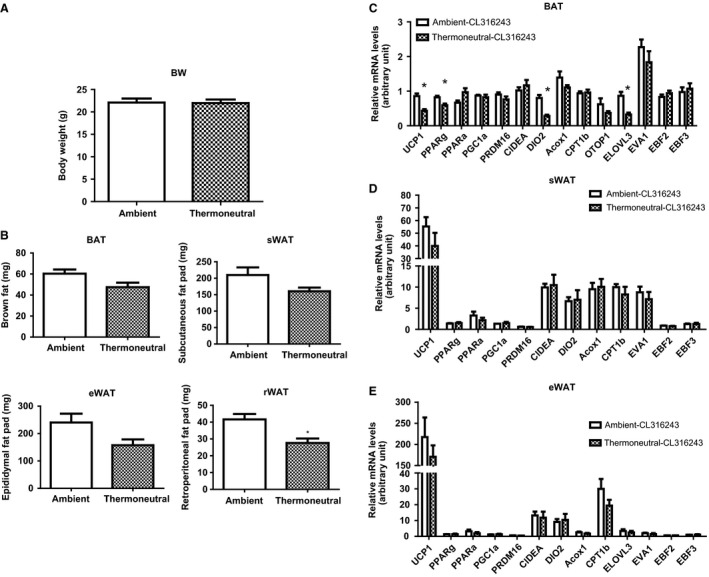
Thermoneutrality does not affect body weight, fat mass, or thermogenic gene expression after *β*‐agonist CL316243 administration. Injection of the *β*‐agonist CL316243 does not change body weight (A) and has a tendency to reduce fat mass (B) in ambient (22°C) and thermoneutral (30°C) mice. Injection of the *β*‐agonist CL316243 has a tendency to reduce the mRNA expression of few thermogenic genes in BAT (C), but does not change the expression of the thermogenic genes in WAT depots (D and E) in ambient (22°C) and thermoneutral (30°C) mice. Eight‐week‐old male mice fed with chow diet were raised at 22°C or 30°C, and were injected with the *β*‐agonist CL316243 (1 mg/kg body weight) for 7 days. mRNA levels in different fat pad were expressed as fold change normalized to ambient temperature (22°C) housed vehicle‐injected samples. All data are expressed as mean ± SEM,* n* = 6. *P* < 0.05 versus Ambient control. BAT, brown adipose tissue; sWAT, subcutaneous white adipose tissue; eWAT, epididymal white adipose tissue.

The thermogenic program in brown and beige adipocytes is mainly regulated by the sympathetic nervous system (SNS) (Himms‐Hagen 1989; Guerra et al. 1998; Xue et al. 2005). We reasoned that if the impaired thermogenic program by thermoneutrality is due to suppression of sympathetic output, the mice housed under the thermoneutral condition would be equally responsive to exogenous *β*3‐adrenergic agonists as the mice housed under the normal ambient condition (or even more responsive for the thermoneutral animals due to enhanced sensitization by lack of persistent stimulation of sympathetic nerve activity). Eight‐week‐old male mice housed at 22°C or 30°C were injected with the *β*3‐agonist CL‐316243 (1 mg/kg body weight) or saline for 7 days to induce adaptive thermogenesis. Indeed, there was no difference in body weight between the ambient and thermoneutral groups (Fig. 6A). The thermoneutral mice treated with CL‐316243 showed a tendency of decrease in fat mass (Fig. 6B). Compared with vehicle injected group, *β*3‐agonist injection stimulated thermogenic gene expression in both brown fat and white fat, especially in subcutaneous white fat. To clarify the difference between ambient and thermoneutral temperature, mRNA levels in different fat pad were expressed as fold change normalized to ambient temperature (22°C) housed vehicle injected samples. Although the expression of several thermogenic genes in BAT (Fig. 6C) of the thermoneutral mice treated with CL‐316243 was lower than that of the ambient mice treated with the *β*3 agonist, no difference in the expression of the thermogenic program in WAT was observed between these two groups (Fig. 6D and E).

**Figure 7:**
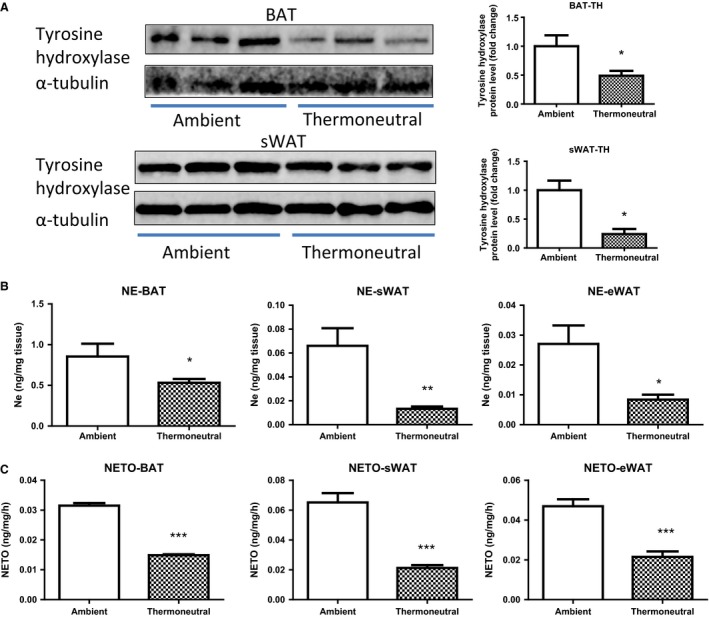
Thermoneutrality suppresses sympathetic nerve activity. (A) Thermoneutrality decreases tyrosine hydroxylase at protein levels in both BAT (upper panel) and WAT (lower panel). (B) Thermoneutrality decreases the NE contents in both BAT and WAT. (C) Thermoneutrality decreases NETO in both BAT and WAT. Mice were raised under either ambient (22°C) or thermoneutral (30°C) conditions. The NE content and NETO measurement in BAT and WAT were conducted as described in the Materials and Methods. All data are expressed as mean ± SEM,* n* = 6; *P* < 0.05 versus Ambient control. BAT, brown adipose tissue; sWAT, subcutaneous white adipose tissue; eWAT, epididymal white adipose tissue; rWAT, retroperitoneal white adipose tissue.

We next directly examined the sympathetic nerve activity in the two groups of mice housed at different thermal conditions. We first measured the expression of tyrosine hydroxylase, the rate‐limiting enzyme catalyzing the synthesis of catecholamines, and found a reduction at protein levels by 50% in BAT and by 70% in WAT of the mice housed under the thermoneutral condition (30°C) (Fig. 7A). The decrease of tyrosine hydroxylase would lead to the repression of sympathetic nerve activity, which can be assessed neurochemically by norepinephrine turnover (NETO) (Bowers et al. 2004; Brito et al. 2007, 2008; Vaughan et al. 2014). We therefore measured the norepinephrine (NE) content and turnover rate (NETO) in fat tissues of both thermal groups. Thermoneutrality decreased the basal NE content by ~40% in BAT (Fig. 7B) and by 60% in WAT depots (Fig. 7C). Similarly, NETO was also reduced by ~50% in both BAT and WAT depots of the thermoneutrally housed mice, suggested an impaired activation of sympathetic nerve under thermoneutrality.

## Discussion

This study was designed to address the hypothesis that thermoneutrality may negatively regulate the thermogenic program and promotes obesity. The plausibility of this hypothesis originates from several prior observations. First, brown/beige adipocytes function to combat obesity via their abilities to generate adaptive thermogenesis in rodents (Feldmann et al. 2009; Seale et al. 2011; Cohen et al. 2014). Recent reports demonstrate that adult humans also possess metabolic active brown fat; the amount of brown fat is inversely correlated with body weight but positively correlated with energy expenditure (Cypess et al. 2009; Marken Lichtenbelt et al. 2009; Virtanen et al. 2009). This important discovery provides new insight into the mechanisms regulating energy homeostasis in adult humans and suggests that increasing functional brown/beige adipocytes in humans is a novel and promising target in treating obesity. Second, with the ability to control the environmental temperature as they desire, humans in modern society are buffered from temperature extremes and spend an increasing amount of time in a thermally comfortable state where energetic demands are minimized (Moellering and Smith 2012). van Marken Linchtenbelt recently indicated that a slighter lower ambient temperature (e.g., 15–16°C) through cold acclimation can induce beige adipocyte appearance and enhance nonshivering thermogenesis in humans, which may play an important role in the development of obesity (van der Lans et al. 2013; Lichtenbelt et al. 2014). However, much is unknown about how the thermogenic program of brown/beige adipocyte thermogenic is regulated by the thermoneutral condition, where loss of heat to the environment is marginal and the energy demanded by regulatory thermogenesis against cold is supposedly suppressed (Lichtenbelt et al. 2014). Since the thermoneutral temperature is within the thermal comfort zone of human dwellings in daily life (Lichtenbelt et al. 2014), this study aims to address the impact of thermoneutrality (30°C) on brown/beige adipocyte's thermogenic program and obesity.

Thermoneutral conditions have been recently used in studies related to metabolism (Speakman and Keijer 2012; Suarez‐Zamorano et al. 2015; Tian et al. 2015). For small rodents (e.g., mouse), the ambient temperature at 22°C represents a significant thermal stress, which requires extra energy expenditure for adaptive thermogenesis to defend body temperature. Previous studies found that mice maintained at the ambient temperature of 22°C have a higher thermogenic activity, which requires about 30% of the calories from food (James and Trayhurn 1981). This extra energy for thermogenesis contributes to the total energy expenditure of the mice (Chaffee and Roberts 1971; Cannon and Nedergaard 2004; Golozoubova et al. 2004), which may ultimately complicate the outcomes of metabolic studies (Kozak and Anunciado‐Koza 2008; Feldmann et al. 2009; Overton 2010; Cannon and Nedergaard 2011). Therefore, the thermoneutral condition, where thermal stress is reduced to minimum, have been used to study metabolism (Keith et al. 2006; Feldmann et al. 2009; McAllister et al. 2009; Hansen et al. 2010; Johnson et al. 2011). We found that thermoneutrality decreases the thermogenic program in both BAT and WAT due to lack of thermal stress. The decreased thermogenesis presumably reduces the overall energy expenditure, which may subsequently demand less energy intake. Diminished energy for thermogenesis coupled with less energy intake may explain the same body weight of the thermoneutrally housed mice. Indeed, given the equal energy intake in the pair‐feeding study, the thermoneutrally housed mice gain 15% more weight than the ambient mice pair‐fed with the same amount of food. The study also suggest that the energy demanded for adaptive thermogenesis to meet the difference of the temperature between ambient and thermoneutral condition may contribute to 15% of body weight gain. However, we found that thermoneutrality decreases the thermogenic program and promotes obesity in HF‐fed mice. The body weight gain in the thermoneutrally housed mice on HF diet is quite significant given the fact that they eat less and that the mouse strain we used is A/J, an obesity‐resistant strain. A previous study suggested that diet‐induced obesity in thermoneutrally housed mice may not result from the changes in metabolic parameters (Hoevenaars et al. 2014). However, that study only examined muscle and liver but not brown or white adipose tissue, which is the major organ for adaptive thermogenesis. Instead, we found a significant suppression of the thermogenic program in both BAT and WAT of the thermoneutrally housed mice, which may at least partially explain the body weight difference. However, several questions still remain. For one, it appears that thermoneutrality exerts differential effect on body weight in HF‐fed and chow‐fed mice. Although both chow‐and HF‐fed mice housed under thermoneutrality had less food intake to meet the decreased energy demanded for adaptive thermogenesis, HF‐fed mice gained weight while chow‐fed did not. To explain the discrepancy, we speculate that high‐fat diet may further suppress the thermogenic program beyond thermoneutrality, resulting in much less energy expenditure that decreased food intake cannot compensate for. This also indicates that western diets coupled with thermoneutrality (e.g., high indoor temperature), two common environmental factors in developed countries, could create a worst‐case scenario for humans to develop obesity. In contrast, lowering few degrees of indoor temperature may make a big difference in prevention of obesity by upregulating the thermogenic program. Second, although being obese, the thermoneutral mice fed HF diet did not develop insulin resistance. One explanation is that thermoneutrality may ease the stress hormone (e.g., catecholamine) production or release, which may decrease insulin sensitivity. Our observation is consistent with previous study showing that thermoneutrality improve insulin sensitivity (Jun et al. 2013). Third, we have found the increased fat mass gain in HF‐fed mice maintained at thermoneutrality, which is consistent with several other studies (Goldgof et al. 2014; Hoevenaars et al. 2014), but not others (Castillo et al. 2011; Veniant et al. 2015). One explanation is that we used A/J mice, which is an obesity‐resistant strain with higher thermogenic tone or UCPs expression (Leibowitz et al. 1047; Surwit et al. 1998), therefore, the suppression of adaptive thermogenesis under thermoneutrality might render them prone to diet‐induced obesity, which is also suggested by the fat pad weight results under ambient temperature (increased tendency of both brown and white fat pad weight under thermoneutral condition). Another explanation might be the different acclimation time, the mice used in this study were bred at thermoneutral temperature, which provided the maximum acclimating time, and the possible effect on sympathetic nervous system development, other studies which showed the positive body weight difference on high‐fat feeding mice also chose long acclimating time for over 2 weeks (Goldgof et al. 2014; Hoevenaars et al. 2014).

Both BAT and WAT are heavily innervated by the sympathetic nerves, activation of which leads to upregulation of the thermogenic program and energy dissipation. We therefore examined the impact of thermoneutrality on the efferent activity of the sympathetic nervous system and the response of brown/beige adipocytes to the *β*‐adrenergic activation. We found that administration of the *β*‐adrenergic receptor agonists (CL316243) had no effect on body weight and even had a tendency to decrease fat mass in the thermoneutrally housed mice. These data suggest that thermoneutrality did not alter the *β*‐adrenergic receptor or downstream signaling and might even sensitize the signaling due to lack of persistent sympathetic activation. On the other hand, we indeed found that thermoneutrality inhibited tyrosine hydroxylase expression, NE contents, and NETO in both BAT and WAT, suggesting a downregulation of sympathetic nerve activity. This may largely explain the diminished thermogenic program and increased adiposity observed in the thermoneutrally house mice.

In summary, we found that thermoneutrally housed mice fed chow diet maintained the same body weight despite eating less, suggesting a decreased efficiency of energy storage. This was accompanied by suppressed expression of the thermogenic program in both BAT and WAT of the thermoneutrally housed mice. Given the same energy intake in a pair‐feeding experiment, these thermoneutrally housed mice gained 15% of more weight, suggesting that the thermoneutrally repressed energy intake contributes to the 15% of the weight gain. Moreover, thermoneutrality, despite inhibiting food intake, increased body weight of mice fed with HF diet. The weight gain was associated with decreased expression of the thermogenic program in both BAT and WAT of the thermoneutrally housed mice. Further, thermoneutrality suppressed sympathetic nerve activity by inhibiting tyrosine hydroxylase expression, NE contents, and NETO, resulting in downregulation of the thermogenic program and promotion of obesity. We conclude that thermoneutrality may negatively regulate sympathetic drive and adaptive thermogenesis, leading to increased adiposity in mice.

## Conflict of Interest

None declared.
